# Biological screening of selected Pacific Northwest forest plants using the brine shrimp (*Artemia salina*) toxicity bioassay

**DOI:** 10.1186/s40064-016-2145-1

**Published:** 2016-04-23

**Authors:** Yvette M. Karchesy, Rick G. Kelsey, George Constantine, Joseph J. Karchesy

**Affiliations:** Wood Science and Engineering, Oregon State University, Corvallis, OR 97331 USA; USDA Forest Service, Pacific Northwest Research Station, Corvallis, OR 97331 USA; College of Pharmacy, Oregon State University, Corvallis, OR 97331 USA

**Keywords:** Brine shrimp lethality, *Artemia salina*, Methanol extracts, Bioactivity

## Abstract

The brine shrimp (*Artemia salina*) bioassay was used to screen 211 methanol extracts from 128 species of Pacific Northwest plants in search of general cytotoxic activity. Strong toxicity (LC_50_ < 100 µg/ml) was found for 17 extracts from 13 species, with highest activity observed for *Angelica arguta* roots at <10 µg/ml. Notably, four species of cedar trees and one of juniper in the family Cupressaceae dominated this group with LC_50_ for heartwood extracts ranging from 15 to 89 µg/ml. Moderate toxicity (LC_50_ 100–500 µg/ml) was found in 38 extracts from 27 species, while weak toxicity (LC_50_ 500–1000 µg/ml) was detected for 17 extracts in 16 species. There were 139 extracts from 99 species that were non-toxic (LC_50_ > 1000 µg/ml). Our subsequent studies of conifer heartwoods with strong activity confirm the assay’s value for identifying new investigational leads for materials with insecticidal and fungicidal activity.

## Background

The forests and rangelands of Washington and Oregon are diverse ecosystems ranging from the temperate rainforests of the Olympic Peninsula in Washington to the semiarid shrub-steppe of southeastern Oregon (Franklin and Dyrness 1988). Across this region, fir, pine and cedar species are basic foundations to industries producing lumber and structural wood products. Native Americans have long used many forest plants for foods, medicines and handmade materials to improve daily life (Gunther 1973; Forlines et al. 1992). There remains an interest in the herbal remedies (Moore 1993), and many of the plants still have potential for development of new, natural sources of medicines and insecticides.

The brine shrimp toxicity bioassay is a simple method of screening crude plant extracts for cytotoxicity (Meyer et al. 1982; McLaughlin et al. 1991) and is an indicator of potential antitumor, insecticidal, and fungicidal activity (Michael et al. 1956; Harwig and Scott 1971; McLaughlin et al. 1998). The mode of action causing toxicity is unknown, but the results typically correlate with more specific bioactivity tests. The brine shrimp bioassay has also been used to guide the isolation of bioactive compounds, testing of water quality, and detection of fungal toxins (Nguta et al. 2011; Arcanjo et al. 2012; Gadir 2012). This method is an attractive pre-screen for such activities as it is relatively simple and inexpensive to test large numbers of crude plant extracts in a relatively short time. Most surveys of this type have been carried out on traditional medicinal plants of various cultures from around the world (Pimentel et al. 2002; Krishnarajua et al. 2005; Rahman et al. 2008; Moshi et al. 2010; Ved et al. 2010; Bussmann et al. 2011; Nguta et al. 2011; Oryema et al. 2011; Arcanjo et al. 2012; Gadir 2012; Nguta et al. 2012; Biradi and Hullatti 2014; Khatun et al. 2014). A few studies have targeted forest and savannah plants (Horgen et al. 2001; Adouom 2009; Rizwana et al. 2010; Soonthornchareonnon et al. 2012; Ravikumar et al. 2014).

In this paper we report survey results for some forest plants from the Pacific Northwest to gain a preliminary understanding of which ones may merit further, more specific testing with potential for developing new medicines and pesticides to benefit future generations.

## Methods

### Plant materials

Plants were collected during their active growing seasons in western Washington, western and central Oregon. Voucher specimens were deposited at the Oregon State University Herbarium.

### Preparation of extracts

Plant materials were air-dried, ground and then extracted at room temperature for 48 h with methanol. The methanol was analytical grade and freshly distilled prior to use. Extracts were evaporated under vacuum on a rotary evaporator and the residue briefly freeze dried under high vacuum to remove traces of solvent and water, then stored at −20 °C until tested.

### Brine shrimp toxicity bioassay

Bioassays of the crude extracts were carried out as described by Meyer et al. (1982) and McLaughlin et al. (1991) on freshly hatched brine shrimp (*Artemia salina* Leach). Triplicate samples of each extract were tested initially at concentrations of 10, 100 and 1000 ppm (µg/mL) in vials containing 5 mL of brine solution and 10 shrimp. Survivors were counted after 24 h and the median lethal concentration (LC_50_) with 95 % confidence intervals calculated using Probit Analysis.

## Results

**Fig. 1:**
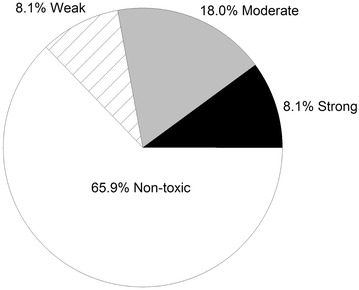
The percentage of extracts within the four categories of cytotoxic activity

Results of the brine shrimp cytotoxicity screening are shown in Table 1. Extracts with LC_50_ values >1000 µg/ml are considered non-toxic (Meyer et al. 1982). Values between 500 and 1000 µg/ml are considered weakly toxic, those between 100 and 500 µg/ml as moderately toxic, and those <100 µg/ml as strongly toxic (Nguta et al. 2012). A total of 211 crude methanol extracts from 128 species, 116 genera, and 49 families are represented. Strong cytotoxic activity was found in 17 extracts from 13 species (Table 2), moderate toxicity in 38 extracts from 27 species, weak activity for 17 extracts in 16 species, and 139 non-toxic extracts from 99 species. The proportions of all extracts by activity category are shown in Fig. 1.

**Table 1:** Brine shrimp toxicity at 24 h exposure to plant extracts

Plant family and species	Common name	Part used	LC_50_ (µg/ml)	95 % CI
Aceraceae				
*Acer circinatum*	Vine maple	Bark	>1000	
		Leaves	>1000	
*Acer macrophyllum*	Big leaf maple	Bark	>1000	
		Catkins	>1000	
Adoxaceae				
*Sambucus nigra subsp. caerulea*	Blue elderberry	Bark	>1000	
		Berries	>1000	
*Sambucus racemosa*	Red elderberry	Bark	>1000	
*Viburnum ellipticum*	Oregon viburnum	Leaves	>1000	
Amaranthaceae				
*Amaranthus retroflexus*	Pigweed	Aerial parts	>1000	
Apocynaceae				
*Apocynum androsaemifolium*	Spreading dogbane	Aerial parts	88	55–141
Araceae				
*Lysichiton americanus*	Skunk cabbage	Flowers	>1000	
		Leaves	>1000	
		Roots	>1000	
Araliaceae				
*Oplopanax horridum*	Devil’s club	Berries-green	338	292–573
		Berries-red	239	187–279
		Leaves	>1000	
		Petioles	237	153–372
		Root bark	21	13–32
		Stem bark	35	23–51
Aristolochiaceae				
*Asarum caudatum*	Wild ginger	Aerial parts	565	364–918
Aquifoliaceae				
*Ilex aquifolium*	Holly	Leaves	>1000	
Berberidaceae				
*Berberis aquifolium*	Tall Oregon grape	Berries green	305	245–352
		Berry stems	>1000	
		Flower heads	608	404–4630
*Berberis nervosa*	Cascade Oregon grape	Leaves	>1000	
		Roots	>1000	
*Berberis repens*	Low Oregon grape	Leaves	>1000	
Betulaceae				
*Alnus rubra*	Red alder	Bark	>1000	
		Leaves	>1000	
*Corylus cornuta*	Hazelnut	Bark	>1000	
Boraginaceae				
*Mertensia paniculata*	Tall bluebell	Aerial parts	>1000	
*Myosotis laxa*	Small flowered forget-me-not	Aerial parts	>1000	
*Symphytum officinale*	Comfrey	Aerial parts	>1000	
Caprifoliaceae				
*Lonicera involucrata*	Black twin-berry	Leaves	>1000	
		Bark	>1000	
*Symphoricarpos albus*	Snowberry	Berries	>1000	
		Leaves	>1000	
Chenopodiaceae				
*Sarcocornia perennis*	Pickleweed	Leaves	>1000	
Compositae (Asteraceae)				
*Achillea millefolium*	Yarrow	Aerial parts	565	364–918
		Leaves only	300	216–402
		Seeds	>1000	
*Ambrosia chamissonis*	Silver burweed	Aerial parts	>1000	
*Anaphalis margaritacea*	Pearly everlasting	Aerial parts	808	403–2800
*Antennaria geyeri*	Pussy toes (Geyer)	Aerial parts	>1000	
*Anthemis cotula*	Dog fennel	Aerial parts	246	182–320
		Roots	>1000	
*Bellis perennis*	Bellis (English daisy)	Aerial parts	454	282–760
*Centaurea* x*moncktonii*	Meadow	Aerial parts	277	203–355
	knapweed	Roots	109	96–152
*Centaurea solstitialis*	Yellow star-	Aerial parts	>1000	
	thistle	Roots	693	423–1349
*Centaurea stoebe subsp. micranthos*	Spotted knapweed	Aerial parts	>1000	
		Roots	87	56–135
*Chrysothamnus viscidiflorus*	Rabbit brush (Green)	Aerial parts	>1000	
*Cichorium intybus*	Chicory	Aerial parts	>1000	
*Cirsium vulgare*	Bull thistle	Aerial parts	>1000	
*Conyza canadensis*	Horseweed	Aerial parts	159	96–267
*Ericameria nauseosa*	Rabbit brush (Gray)	Aerial parts	579	360–1006
*Eriophyllum lanatum*	Woolly sunshine	Aerial parts	>1000	
*Grindelia integrifolia*	Gumweed	Aerial parts	173	107–276
		Roots	99	75–116
*Hypochaeris glabra*	Cat’s ear	Aerial parts	>1000	
*Lapsana communis*	Nipplewort	Aerial parts	>1000	
*Leucanthemum vulgare*	Oxeye daisy	Aerial parts	16	10–25
		Roots	164	139–183
*Madia sativa*	Tarweed	Aerial parts	>1000	
*Matricaria discoidea*	Pineapple weed	Aerial parts	192	160–208
*Senecio jacobaea*	Tansy ragwort	Aerial parts	>1000	
*Solidago canadensis*	Canada goldenrod	Aerial parts	827	458–2214
*Sonchus asper*	Prickly sow	Leaves	>1000	
	thistle	Roots	>1000	
*Symphyotrichum subspicatum*	Douglas aster	Aerial parts	>1000	
*Tanacetum vulgare*	Common tansy	Aerial parts	62	39–93
*Tragopogon porrifolius*	Salsify	Aerial parts	>1000	
Convolvulaceae				
*Convolvulus arvensis*	Orchard morning glory	Aerial parts	>1000	
Cornaceae				
*Cornus nuttallii*	Dogwood	Bark	>1000	
Cupressaceae				
*Callitropsis nootkatensis*	Yellow-cedar	Foliage	42	27–65
		Heartwood	89	53–114
		Outer Bark	693	423–1349
		Inner Bark	15	8–24
		Sapwood	>1000	
*Calocedrus decurrens*	Incense cedar	Heartwood	55	35–80
		Sapwood	>1000	
*Cedrus deodara* ^1^	Deodar cedar	Heartwood	15	9–24
		Sapwood	36	30–39
*Chamaecyparis lawsoniana*	Port Orford cedar	Heartwood	31	23–39
×*Hesperotropsis leylandii*	Leyland cypress	Heartwood	118	81–161
		Sapwood	>1000	
*Juniperus occidentalis*	Juniper	Berries	>1000	
	(Western)	Leaves	>1000	
		Heartwood	66	56–77
		Inner Bark	>1000	
		Outer Bark	>1000	
		Sapwood	189	116–338
Elaeagnaceae				
*Shepherdia canadensis*	Soapberry	Berries	387	255–571
		Leaves	>1000	
		Leaves with twigs	>1000	
		Outer Bark	314	174–662
Ericaceae				
*Arbutus menziesii*	Pacific madrone	Inner Bark	>1000	
		Red berries	>1000	
*Arctostaphylos columbiana*	Hairy manzanita	Bark	>1000	
		Leaves	>1000	
*Arctostaphylos patula*	Green leaf manzanita	Aerial parts	>1000	
*Arctostaphylos uva*-*ursi*	Kinnikinnick	Berries-red Leaves/stems	>1000	
			>1000	
*Chimaphila umbellata*	Prince’s pine	Aerial parts	155	131–177
		Stems	126	86–170
*Gaultheria shallon*	Salal	Leaves	>1000	
*Rhododendron macrophyllum*	Pacific rhododendron	Bark	>1000	
		Leaves	>1000	
Fagaceae				
*Quercus garryana*	White oak	Galls	>1000	
		Heartwood	301	195–468
		Inner Bark	>1000	
		Leaves	>1000	
Fumariaceae				
*Dicentra formosa*	Wild bleeding heart	Aerial parts	>1000	
Geraniaceae				
*Geranium dissectum*	Cut-leaf geranium	Aerial parts	>1000	
Iridaceae				
*Iris tenax*	Oregon iris	Aerial parts	>1000	
Labiatae				
*Prunella vulgaris*	Heal all; Self-heal	Aerial parts	>1000	
*Stachys cooleyae*	Cooley’s hedge nettle (False stinging nettle)	Aerial parts	>1000	
Lauraceae				
*Umbellularia californica*	Oregon myrtle	Heartwood	363	255–488
		Sapwood	>1000	
Leguminosae				
*Cytisus scoparius*	Scotch broom	Aerial parts	>1000	
*Dalea ornata*	Prairie clover	Aerial parts	157	95–257
		Roots	313	121–1632
*Robinia pseudoacacia*	Black locust	Heartwood	>1000	
*Trifolium pratense*	Red clover	Aerial parts	>1000	
Liliaceae				
*Camassia quamash*	Camas	Aerial parts	212	150–952
		Flowers	272	148–583
		Leaves	446	256–905
*Prosartes smithii*	Smith’s fairy bell	Aerial parts	>1000	
Malvaceae				
*Malva neglecta*	Dwarf mallow	Aerial parts	>1000	
Nyctaginaceae				
*Abronia latifolia*	Yellow sandverbena	Aerial parts	>1000	
Onagraceae				
*Chamerion angustifolium*	Fireweed	Aerial parts	>1000	
Oxalidaceae				
*Oxalis oregana*	Oxalis	Aerial parts	281	268–298
Pinaceae				
*Abies grandis*	Grand-fir	Needles (new)	>1000	
		Needles (old)	>1000	
*Picea sitchensis*	Sitka spruce	Needles	>1000	
*Pinus monticola*	Western white	Bark	>1000	
	pine	Needles	504	397–662
*Pinus ponderosa*	Ponderosa pine	Bark	>1000	
		Heartwood	107	69–166
		Needles	>1000	
		Sapwood	>1000	
*Pseudotsuga menziesii*	Douglas-fir	Cones-green	>1000	
		Heartwood	663	422–1153
		Needles	>1000	
		Outer bark	>1000	
		Sapwood	>1000	
*Tsuga heterophylla*	Western	Cones-green	>1000	
	hemlock	Needles	>1000	
		Sapwood	>1000	
Plantaginaceae				
*Plantago spp.*	Plantain	Aerial parts	>1000	
Polygonaceae				
*Rumex* spp.	Dock	Roots	923	822–1537
Polypodiaceae				
*Polypodium glycyrrhiza*	Licorice fern	Roots	>1000	
*Polystichum munitum*	Sword fern	Leaves	>1000	
		Roots	>1000	
*Pteridium aquilinum*	Bracken fern	Roots	>1000	
Portulacaceae				
*Claytonia sibirica*	Siberian miners’ lettuce	Aerial parts	>1000	
Primulaceae				
*Trientalis latifolia*	Western starflower	Aerial parts	539	430–627
Ranunculaceae				
*Clematis vitalba*	Clematis	Aerial parts	>1000	
*Delphinium trolliifolium*	Delphinium	Aerial parts	304	190–489
*Ranunculus occidentalis*	Western buttercup	Aerial parts	>1000	
*Ranunculus repens*	Creeping buttercup	Aerial parts	>1000	
Rhamnaceae				
*Rhamnus purshiana*	Cascara	Bark	393	237–698
		Leaves	247	186–667
Rosaceae				
*Aruncus dioicus*	Goat’s beard	Flowers	>1000	
		Leaves	>1000	
		Roots	>1000	
*Crataegus douglasii*	Black hawthorn	Berries-green	>1000	
		Leaves	>1000	
*Holodiscus discolor*	Ocean spray	Bark	>1000	
		Flowers	>1000	
		Leaves	>1000	
*Malus fusca*	Crabapple	Bark	>1000	
*Oemleria cerasiformis*	Indian-plum	Bark	>1000	
		Stems + leaves + berries	>1000	
*Potentilla pacifica*	Pacific silverweed	Leaves	632	298–2309
*Prunus* spp.	Cherry	Leaves	>1000	
		Inner Bark	>1000	
		Outer Bark	490	354–614
*Purshia tridentata*	Bitter-brush	Leaves	870	533–1857
		Roots	691	545–884
		Seeds	144	101–192
*Rosa nutkana*	Nootka rose	Leaves	>1000	
		Stems	>1000	
*Rubus parviflorus*	Thimbleberry	Leaves	>1000	
*Rubus spectabilis*	Salmonberry	Bark	>1000	
		Leaves	>1000	
*Rubus ursinus*	Blackberry (trailing)	Aerial parts	>1000	
*Sorbus scopulina*	Mountain ash	Berries	318	308–328
		Leaves	>1000	
*Spiraea douglasii*	Spirea	Aerial parts	>1000	
Rubiaceae				
*Galium aparine*	Cleavers	Aerial parts	>1000	
Salicaceae				
*Populus* spp.	Cottonwood	Outer Bark	>1000	
Saxifragaceae				
*Tellima grandiflora*	Fringecup	Aerial parts	>1000	
Scrophulariaceae				
*Digitalis purpurea*	Foxglove	Aerial parts	>1000	
*Verbascum thapsus*	Common mullein	Aerial parts	>1000	
		Roots	>1000	
Solanaceae				
*Solanum nigrum*	Black nightshade	Aerial parts	662	422–1153
Taxaceae				
*Taxus brevifolia*	Pacific yew	Heartwood	>1000	
Taxodiaceae				
*Sequoiadendron giganteum*	Giant sequoia	Needles	713	580–878
		Heartwood	206	166–246
Umbelliferae				
*Angelica arguta*	Sharptooth angelica	Aerial parts	123	94–371
		Roots	<10	–^2^
*Daucus carota*	Queen Anne’s lace	Aerial parts	>1000	
*Foeniculum vulgare*	Fennel	Aerial parts	>1000	
*Heracleum maximum*	Cow parsnip	Roots	249	167–384
		Umbels	404	307–496
*Oenanthe sarmentosa*	Pacific water parsley	Aerial parts	76	48–117
Urticaceae				
*Urtica dioica*	Stinging nettle	Aerial parts	>1000	
		Roots	>1000	

^1^Endemic to the Indian subcontinent, collected from a tree farm in Oregon

^2^10 µg/ml was the lowest concentration tested with mean mortality at 90 %

**Table 2:** Plant species and tissues with strong, <100 µg/ml LC_50_, brine shrimp toxicity at 24 h exposure to plant extracts

Species	Part used	LC_50_ (µg/ml)	95 % CI
*Apocynum androsaemifolium*	Aerial parts	88	55–141
*Oplopanax horridum*	Root bark	21	13–32
	Stem bark	35	23–51
*Centaurea stoebe subsp.*	Roots	87	56–135
*micranthos*			
*Grindelia integrifolia*	Roots	99	75–116
*Leucanthemum vulgare*	Aerial parts	16	10–25
*Tanacetum vulgare*	Aerial parts	62	39–93
*Callitropsis nootkatensis*	Foliage	42	27–65
	Heartwood	89	53–114
	Inner bark	15	8–24
*Calocedrus decurrens*	Heartwood	55	35–80
*Cedrus deodara* ^1^	Heartwood	15	9–24
	Sapwood	36	30–39
*Chamaecyparis lawsoniana*	Heartwood	31	23–39
*Juniperus occidentalis*	Heartwood	66	56–77
*Angelica arguta*	Roots	<10	–^2^
*Oenanthe sarmentosa*	Aerial parts	76	48–117

^1^Endemic to the Indian subcontinent, collected from a tree farm in Oregon

^2^10 µg/ml was the lowest concentration tested with mean mortality at 90 %

## Discussion

There were more than twice as many extracts with moderate activity than there were with strong activity. Moderately active extracts need not be dismissed as unimportant, since Bussmann et al. (2011), Nguta et al. (2012) and others have noted that toxicity can vary significantly due to harvest time, collection location, plant organ or tissue, and solvent used for extraction. Alcohol or organic solvent extracts are often more toxic than aqueous ones, but not always. Extracts from genera and species with the strongest bioactivity can also exhibit a wide range in their levels of activity for the same reasons, thus varying among experiments and research groups. Given this natural variability and our extensive list of genera and species we decided not to attempt cross comparing levels of activity with those observed by others, as it is beyond the scope of this report.

Tissues identified with LC_50_ < 100 µg/ml cytotoxicity have served us as leads for further studies of bioactive extracts and compounds from heartwoods of yellow, incense, and Port-Orford cedars, and western juniper against mosquitoes (*Aedes aegypti*), ticks (*Ixodes scapularis*), fleas (*Xenopsylla cheopis*) or microbes influencing animal and forest health (Johnston et al. 2001; Panella et al. 2005; Dietrich et al. 2006; Manter et al. 2006, 2007; Dolan et al. 2007, 2009). It is worthwhile noting that three of the compounds in yellow or incense cedar heartwoods have different modes of action than other commercially available mosquito adulticides currently in use (McAllister and Adams 2010). New modes of action are particularly relevant in the search for compounds to overcome resistance to existing pesticides.

## Conclusion

Natural products from Pacific Northwest forest resources can offer alternative biocides and repellent compounds with activities comparable to synthetic pesticides for control of arthropods of public health concern and forest microbial pathogens. Other bioactive extracts from our brine shrimp screening need to be investigated further. In addition, other forest plants from this region need to be pre-screened by this method as well to provide a more complete understanding of the potential value for all our forest and rangeland resources.
